# Supplementary material for: Lytic Polysaccharide Monooxygenase from Talaromyces amestolkiae with an Enigmatic Linker-like Region: The Role of This Enzyme on Cellulose Saccharification
Source: Int J Mol Sci. 2021 Dec 19;22(24):13611. doi: 10.3390/ijms222413611 (PMC8703934; doi:10.3390/ijms222413611)
Supplement: Supplementary file 1 [file ijms-22-13611-s001.zip › ijms-1498663-supplementary.pdf]

International Journal of *Molecular Sciences*

MDPI

Article

Lytic Polysaccharide Monooxygenase from *Talaromyces amestolkiae* with an Enigmatic Linker-like Region: The Role of This Enzyme on Cellulose Saccharification

Supplementary Material of "A novel AA9 LPMO of the fungus *Talaromyces amestolkiae*: its potential to improve the saccharification of lignocellulosic residues"

> TamAA9A DNA SEQUENCE

ATGCCTTCCACTAAAGTCGCTGCTGTTTCTGCTATTCTAGCATTGGCCTCCACTGTTGCTGGCCATGGTTATGTGCAAAACATCGTT
ATCGATGGTGAATCGTAAGTAGTGATACCTTTATTGAAGCAAGAGAAATTCTTACAAGTGAATCAGCTACTCTGGATACATTGTGAC
TCAGTTTCCCTACGAGTCCAACCCCCCCGCTGTCATTGGATGGGCAACTACTGCAACTGACTTGGGATTCGTCGATCCCAGTGAGTA
CACGAATGCAGACATCATCTGCCACAAGAATGCCACACCTGGAGCAATTTCCGCTTCAGTCGCCGCAGGAGGTACTGTCGAGCTCCA
GTGGACCACATGGCCCGATAGCCATCACGGTCCCGTCATCAGCTATCTCGCCAACTGCAACGGTAACTGTTCTACCGTGGACAAGAC
TACTCTAGACTTCGTCAAGATTGACGAAGGTGGTCTGATCGACGACACCACCGTCCCAGGTACGTGGGCTTCAGACCAACTTATCGC
CGCCAACAACAGCTGGACTGTAACCATCCCTGATACCATCGCACCTGGAAACTATGTTTTGCGCCACGAAATCATTGCTCTTCACTC
CGCCGGAAACACAGACGGTGCCCAAAACTACCCTCAATGCATCAACTTGGAGATCACTGGCAGCGGAACCGCCAGTCCCTCTGGAAC
CGCTGGTGAAAAGCTGTACACCCCTACTGACCCCGGTATCTTGGTCAACATCTACCAATCCTTGTCGACCTACGTTATTCCCGGACC
GACTCTGTGGAGCGGTGCTGCAACTGACGCTGTTGCCACTGGTTCTGCTACTGCAGTCGCTACGACTGCCGCTGCTTCTGCGACTGC
TACTCCTACCACACTTGTTACCTCTGTCGCATCCGCTACTGACTCGCCTTCAACTGTGGCTCCGGCTTCATCTACCACTGCCACCTC
CGTCGCAACCTCCGTCGCTCCTGCGGTGACTTCCTTCGTCGATGTCGTGACTGTTACTGATGTCGTTACCGTGACCACCGTCATCAC
CACCACTGTCTTTTAA

> TamAA9A PROTEIN SEQUENCE

MPSTKVAAVSAILALASTVAG**H**GYVQNIVIDGESYSGYIVTQFPYESNPPAVIGWATTAT
DLGFVDPSEYTNADIICHKNATPGAISASVAAGGTVELQWTTWPDSHHGPVISYLANCNG
NCSTVDKTTLDFVKIDEGGLIDDTTVPGTWASDQLIAANNSWTVTIPDTIAPGNYVLRHE
IIALHSAGNTDGAQNYPQCINLEITGSGTASPSGTAGEKLYTPTDPGILVNIYQSLSTYV
IPGPTLWSGAATDAVATGSATAVATTAAASATATPTTLVTSVASATDSPSTVAPASSTTA
TSVATSVAPAVTSFVDVVTVTDVVTVTTVITTTVF-

Figure S1. DNA and amino acid sequences of TamAA9A from *T. amestolkiae*. In DNA sequence, intron is indicated in yellow. Signal peptide is underlined. Start and stop transcription codons are marked in red. In protein sequence, signal peptide is marked in blue. Initial histidine is in bold. The underlined part corresponds to the linker-like region of the enzyme.

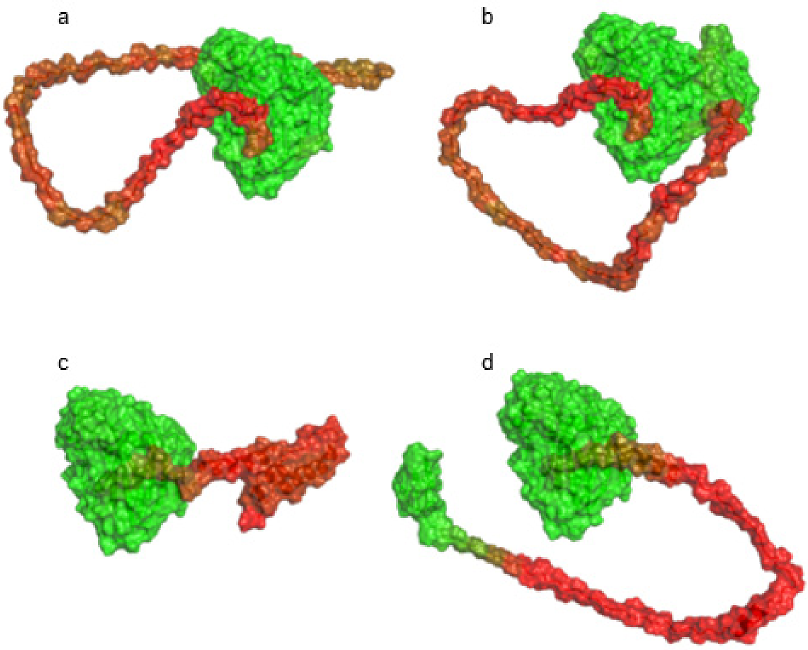

Figure S2. Molecular models of TamAA9Awt or TamAA9A-CBM obtained with AlphaFold2.0 (a and b, respectively) or RoseTTAFold (c and d, respectively), based in the crystal structure of *P. verruculosum* LPMO. Colors show the accuracy of the model from green (high accuracy) to red (poor accuracy).

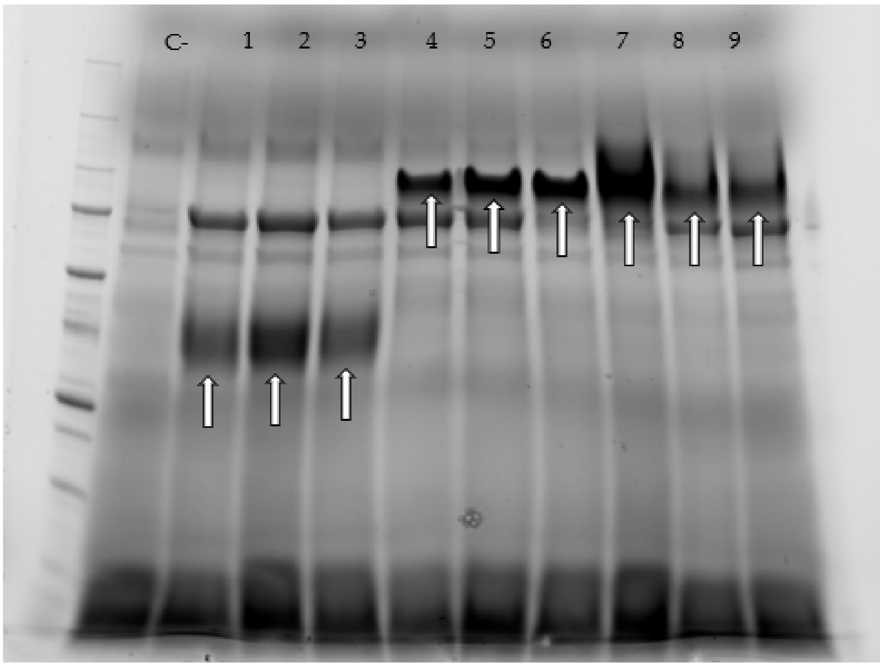

Figure S3. (A) Analysis of the expression of the three LPMO variants by SDS-PAGE. C-, non-transformed *P. pastoris* X-33 strain negative control. Lanes 1, 2 and 3, TamAA9A-CD producing clones. Lanes 4, 5 and 6, TamAA9A-WT producing clones. Lanes 7, 8 and 9, TamAA9A-CBM producing clones.

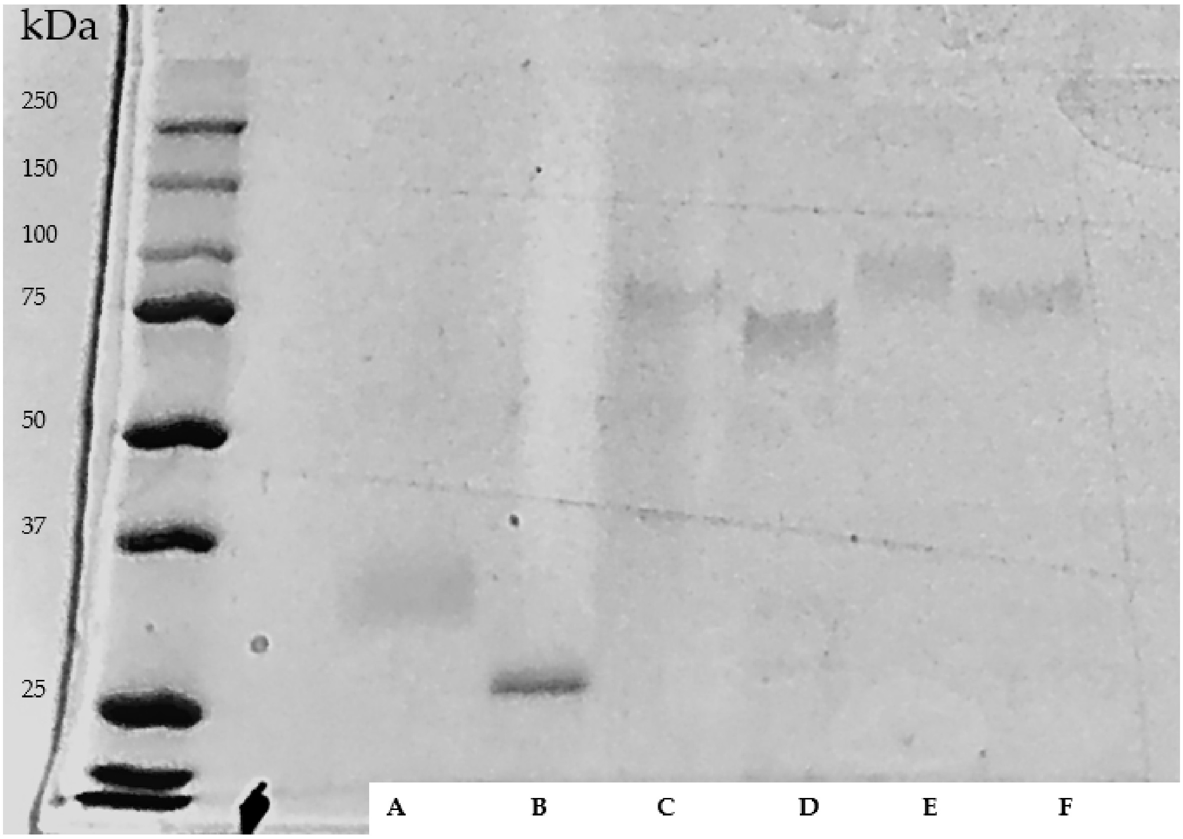

Figure S4. Deglycosylation experiment with the three LPMO variants, analyzed by SDS-PAGE. Only a small decrease in the molecular mass of TamAA9AWT and TamAA9ACBM could be observed. (A: glycosylated TamAA9ACD. B: Deglycosylated TamAA9ACD. C: glycosylated TamAA9AWT. D: Deglycosylated TamAA9AWT. E: glycosylated TamAA9ACBM. F: Deglycosylated TamAA9ACBM).

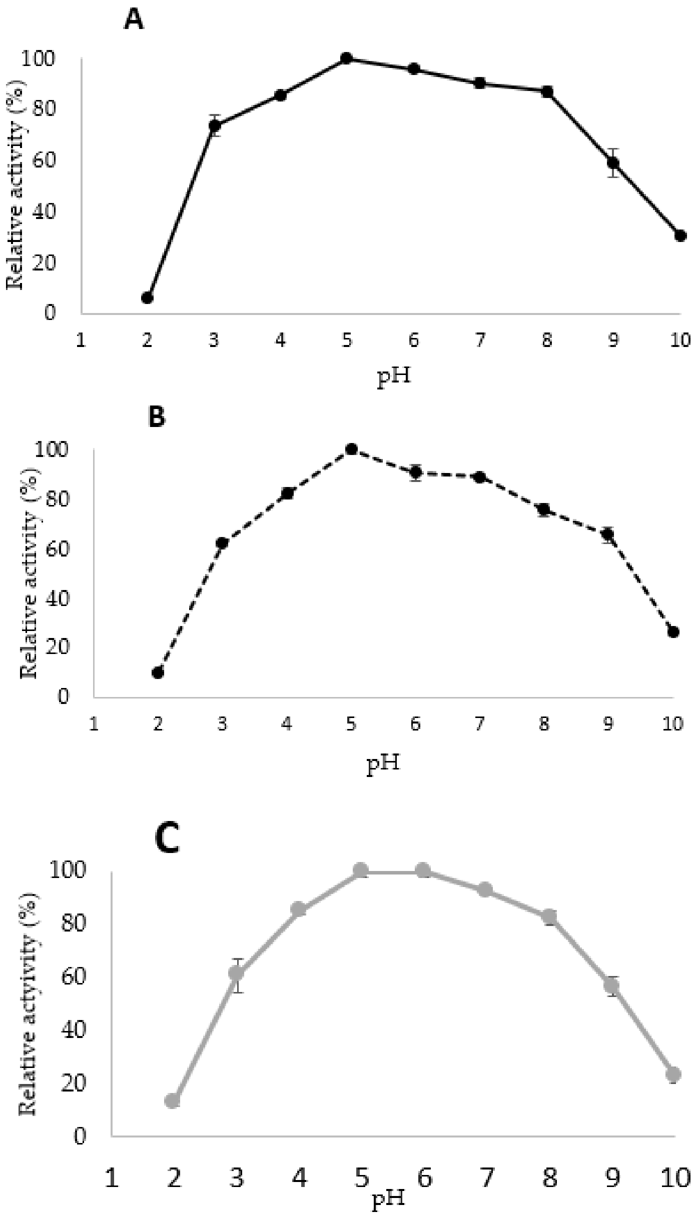

Figure S5. pH stability of TamAA9ACD (A), TamAA9AWT (B) and TamAA9ACBM (C). For pH stability tests, the enzyme was incubated at the appropriate pH in Britton-Robinson buffer, 100 mM at 4° C, during 24 h. After this, enzymes were dialyzed again to pH 6, and standard 2,6-DMP oxidation reaction was carried out. All reactions were performed in triplicate.

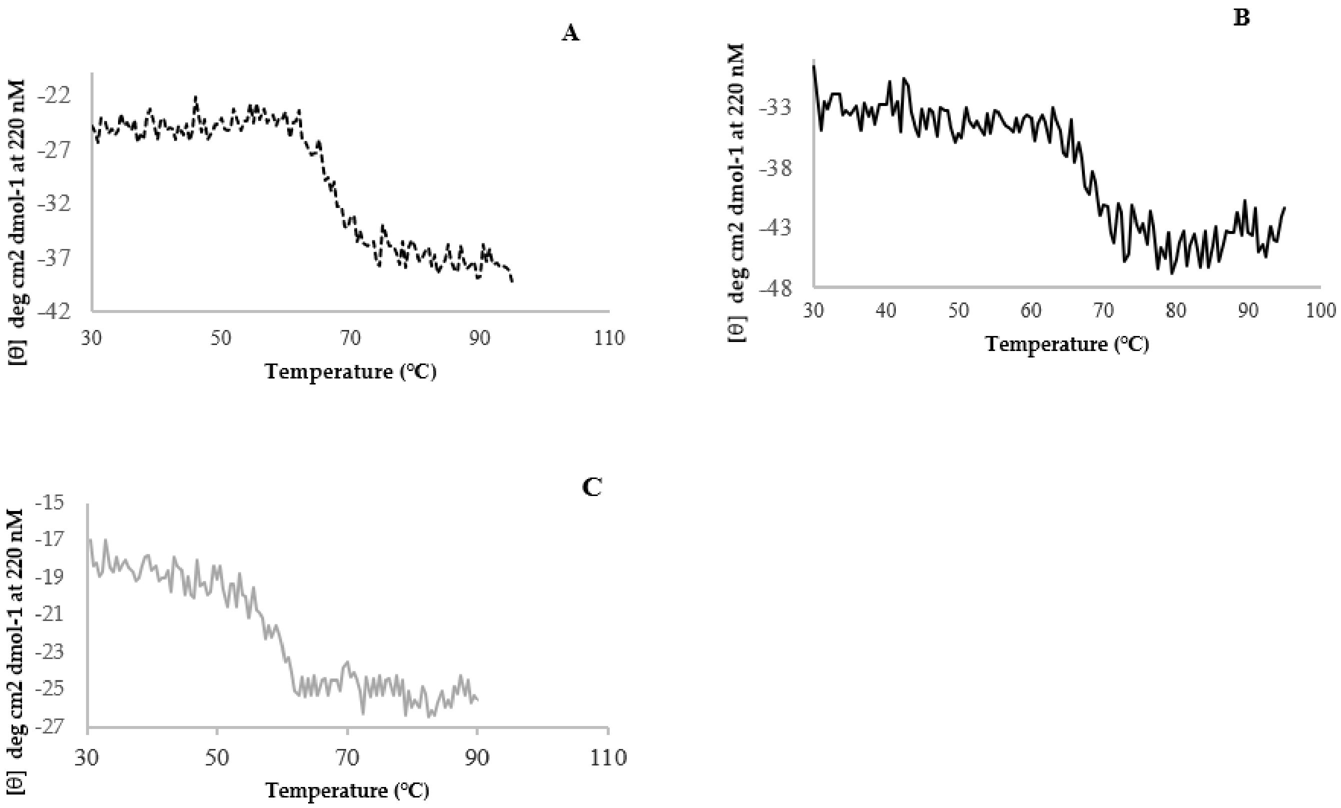

Figure S6. Study of thermostability of TamAA9 variants by circular dicroism. **(A)** TamAA9ACD temperature ramp, **(B)** TamAA9AWT, **(C)** TamAA9ACBM temperature ramp.
